# Partnering with Consumers – Why would I do it? What is it? How do I do it?

**DOI:** 10.1002/jmrs.782

**Published:** 2024-03-26

**Authors:** Jodie Nixon

**Affiliations:** ^1^ Clinical Governance, Risk and Legal Metro South Health Eight Mile Plains Queensland Australia; ^2^ School of Health and Rehabilitation Sciences University of Queensland Brisbane Queensland Australia

## Abstract

Health care and research are increasingly mandating consumer involvement in the planning, design and evaluation of services, quality projects and research. The editorial reviews the Australian progress with accreditation processes in research and provides practical direction in an area that is unfamiliar to many researchers and clinicians.

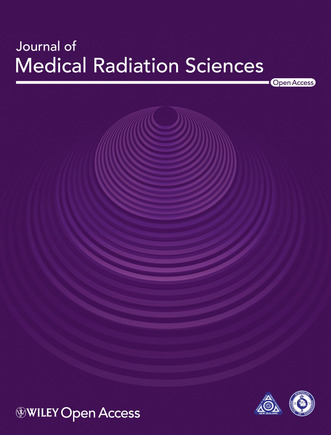

## Partnering with Consumers – Why Would I do it?

Health care and research are increasingly accepting and mandating consumer involvement in the planning, design and evaluation of services.^1^ It can be both exciting and daunting for researchers and clinicians to understand how to include consumers in work that has historically been driven by researchers, clinicians, and funding bodies alone. The original article published in this issue by Brown et al.^2^ describes two separate scenarios where consumer engagement and partnerships have successfully been embedded into a quality improvement project and a research project in the field of Medical Radiation Sciences.^2^ This editorial expands on the mandated requirements for partnering with consumers in Australian health contexts and provides recommendations that will set up future projects for success.

The focus of this editorial is the Australian health context, however, in developing the Australian Governance Framework, it was noted that many countries have attempted to develop systems to improve the oversight of research trials (Table 1). Countries such as Australia, Canada, the United Kingdom (UK) and South Korea primarily have health service systems that have single‐payer systems which enable a more centralised establishment of accreditation and governance systems.^3^ The USA and European Union countries have a variety of health service systems and funding models which have made the adoption of a universal Governance Framework difficult to implement as a national approach.^3^

**Table 1:** Research accreditation for USA, UK, India, and China.

Country	National Accreditation agency	Accreditation for Research Trials	Comments/Reference
United States	The Joint Commission‐ linked with 22,000+ organisations	Agency for Healthcare Research and Quality	No centrally endorsed accreditation. https://www.ahrq.gov
United Kingdom	The United Kingdom Accreditation Service	The National Institute for Health Research	https://www.nihr.ac.uk
India	National Accreditation Board for Hospitals and Healthcare Providers	Local processes only	
China	China central government	Local processes only	Some local provinces are developing accreditation processes

Since May 2023, it is mandatory for all public and private Australian health services involved in clinical trials to be assessed against the National Clinical Trials Governance Framework through an independent accreditation process.^1^ To align with the National Safety and Quality Health Service (NSQHS) Standards, the National Clinical Trials Governance Framework adopts the Clinical Governance Standard (Standard 1) and the Partnering with Consumers Standard (Standard 2), and sets out mandatory actions across five components under the two Standards. The Framework differentiates between the terms ‘trial participant’, ‘patient’ and ‘consumer’ to provide clarity on their involvement. ‘A patient is a person who is receiving care in a health service organisation and a consumer is a person who has used, or may potentially use, health services, or is a carer for a patient using health services’.^1^ ^(p5)^ A trial participant is ‘a patient or healthy volunteer who has been enrolled in a clinical trial’.^1^ ^(p5)^

Actions in the Partnering with Consumers Standard set the expectations for: (i) how clinical governance and quality improvement systems need to be established to ensure that consumers are involved in healthcare planning, design, measurement, and evaluation and (ii) how participants and patients participate as partners in their own care, to the extent that they choose.^1^ The standards are designed to ensure consistency of performance but do not specify how to develop or implement systems, so each organisation or site can develop a system that supports their local context. The challenge for organisations, researchers and clinicians is to ensure that consumers are involved in research and quality improvement from original design through to implementation and evaluation.

## Partnering with Consumers – What is it?

The most widely used tool for understanding how to engage with consumers is the internationally recognised IAP2 (International Association for Public Participation) spectrum.^4^ There are five levels in the participation spectrum (Inform, Consult, Involve, Collaborate and Empower) with increasing impact on decision making as the involvement of consumers moves along the spectrum.^2^, ^4^ Several nationally endorsed frameworks in clinical and research fields use this spectrum as the basis for consumer engagement and participation, including the National Framework for Consumer Involvement in Cancer Control,^5^ the South Australian Health and Medical Research Institute Integrated Consumer Engagement in Health and Medical Research – An Australian framework^6^ and the National Health and Medical Research Council (NHMRC) and Consumers Health Forum of Australia, which in 2016 introduced a *Statement on consumer and community involvement in health and medical research*.^7^ The NHMRC subsequently developed and has published a toolkit for researchers and clinicians with resources to support consumer engagement in research and quality improvement.^8^

The five levels on the participation spectrum are described below with examples of how research or quality projects have applied these in local contexts.

*Inform* – *C*onsumers and communities are kept informed by organisations.^4^, ^5^ An example of this is the Cancer Australia website Cancer Australia (www.canceraustralia.gov.au) which anyone can visit to read the latest news about multiple cancer initiatives,^9^ or the Inside Radiology website (https://insideradiology.com.au) which gives consumers simple information on a variety of clinical radiology procedures and supporting information.^10^ The inform level is a one‐way direction of information flow.

*Consult* – Consumers and communities are consulted on key issues that affect them.^4^, ^5^ This includes research participants or people who complete surveys to help provide researchers and clinicians with direction for service change. An example of the consult level is the Case Study 1 presented in Brown et al.^2^ where the Youth Advisory group and young people undergoing treatment had the opportunity to complete formal surveys. The feedback obtained supported the development of clinical education resources for radiation therapists.^2^ Another example of a consult phase was a study completed by Nixon et al.^11^ which explored what people with cancer and their family members defined as wellness, and what supports were needed to support wellness during the cancer experience. This led to the development of a website with local information and resources (https://pacancerwellness.com).

*Involve* – Consumers and communities are involved in issues/projects together with an organisation.^4^, ^5^ An example of this is the newly established consumer advisory group at the Australian Bragg Centre for Proton Therapy and Research. The story of how this group developed can be read online in the Journal of Medical Radiation Science.^12^ The Bragg Consumer Advisory Group was established in 2023 and comprises 10 members with diverse backgrounds and experiences. The group commenced at the ‘involve’ level of participation, but with the appointment of a consumer‐led chair and co‐chair, and active involvement in decision‐making process the Bragg Consumer Advisory Group is progressing to the ‘collaborate’ level.^4^, ^12^

*Collaborate* – Consumers and the organisation share ownership and accountability for the process and decisions made.^4^, ^5^ This requires commitment on behalf of both parties and, as recommended by multiple researchers,^2^, ^13^ consideration of time, remuneration, and at least two active consumer partners to support each other as peers.^14^ An Australian example of a successful research team is demonstrated by Cox et al.^15^ in a paper titled ‘Learning and growing together’.^15^ This paper reports on a PhD student's project with two academics and four consumer co‐researchers. Outputs from the collaboration included six publications and the development of a co‐produced capability development framework for building successful staff and consumer partnerships in quality improvement for healthcare. The framework is now being used in a health service to develop a co‐produced learning program.^15^, ^16^

*Empower* – Consumers may be asked to lead a project for organisations and make all key decisions.^4^, ^5^ The author is not aware of research activity in Medical Radiation Science or Cancer Services in Australia led solely by a consumer.

## Partnering with Consumers – How do I do it?

Embedding partnering with consumers into research and quality improvement is now not only mandated but also considered the ethical approach that leads to better research and quality outcomes.^1^, ^2^, ^13^, ^15^ Like any skill it can be learnt, but can feel difficult to know where and how to start. There are many resources available and people who are willing to provide support.

Metro South Health is a major health service in Queensland, Australia who services a community of over 1.2 million people.^17^ Over the last 3 years the health service has embarked on an organisational change effort to integrate consumers in the governance of the health service at all levels based on the National Model Clinical Governance Framework which encourages that consumer partnering is at the centre of the five elements required for delivery of safe patient care.^18^, ^19^ Further information to support health services to successfully implement consumer partnering into a Clinical Governance Unit can be found in the paper by Nixon et al.^19^

At an individual level, it is recommended that researchers and clinicians become familiar with the IAP2 spectrum and the level that you may feel comfortable to commence engagement.^4^

Recommendations to support you with the process of partnering with consumers:

1. Reach out to your consumer partner/engagement team. As discussed by Brown et al.^2^ they found varying levels of consumer guidelines across the two major health services where the case study projects were undertaken. If you are unsure how to contact consumer partner/engagement team, ask the local Clinical Governance contact for suggestions on who to approach.
2. Use existing resources – you do not have to reinvent the wheel. Brown et al.^2^ made a strong recommendation if you do not have local consumer supports find other avenues which may be state‐based organisations such as Health Consumers Queensland or Safer Care Victoria.^15^, ^20^
3. Be flexible with timing and consider offering remuneration. Most people who are involved with partnering with consumers are very happy to share their experiences, so that you do not have to repeat the same challenges. Brown et al.^2^ stressed the importance of acknowledging consumers lived experience through the valuing of remuneration, and as a learning shared that next time, they would budget remuneration into their project from commencement. Many organisations and grant opportunities now recommend remuneration. Do not let lack of remuneration be an obstacle. Be transparent whether you have the option or not, and the consumer partner can then make the decision regarding their involvement.
4. Spend the time to build relationships.^2^, ^5^, ^13^, ^15^ The stronger the relationship between the team, the better research and quality outcomes you will achieve.
5. Enjoy the process.

## Conflict of Interest Statement

The author declares no conflcit of interest.

## Data Availability

Data sharing not applicable to this article as no datasets were generated or analysed during the current study.
